# Predictive value of preoperative magnetic resonance imaging structural and diffusion indices for the results of trigeminal neuralgia microvascular decompression surgery

**DOI:** 10.1007/s00234-023-03155-4

**Published:** 2023-05-04

**Authors:** Yang Zhang, Dengbin Sun, Yunjie Xie, Rui Li, Hang Zhao, Zhaoping Wang, Lei Feng

**Affiliations:** 1 Department of Neurosurgery, Jining No. 1 People’s Hospital, Jining, Shandong 272001 People’s Republic of China; 2 Department of Radiology, Jining No. 1 People’s Hospital, Jining, Shandong 272001 People’s Republic of China

**Keywords:** Trigeminal neuralgia, Spinal trigeminal tract, Microvascular decompression, Combined diagnosis

## Abstract

**Purpose:**

To explore the predictive value of preoperative magnetic resonance imaging structural and diffusion indices of the spinal trigeminal tract (SpTV) on the results of microvascular decompression (MVD) in patients with trigeminal neuralgia (TN).

**Methods:**

This retrospective study included patients diagnosed with TN and treated with MVD in the Jining First People’s Hospital between January 2020 and January 2021. The patients were divided into good and poor results groups according to postoperative pain relief. Logistic regression analysis was performed to explore independent risk factors for poor results of MVD, and their predictive value was examined using receiver operating characteristic (ROC) curves.

**Results:**

A total of 97 TN cases were included, 24 cases with a poor result and 73 with a good result. They were comparable in demographic characteristics. Fractional anisotropy (FA) was lower (*P* < 0.001), and radial diffusivity (RD) was higher (*P* < 0.001) in the poor result group compared to the good result group. Patients in the good result group showed a higher proportion of grade 3 neurovascular contact (NVC) (39.7% vs. 16.7%, *P* = 0.001) and a lower RD (*P* < 0.001). The multivariate analysis showed that the RD of SpTV (OR = 0.000016, 95% CI: 0.000–0.004, *P* < 0.001) and NVC (OR = 8.07, 95% CI: 1.67–38.93, *P* = 0.009) were independently associated with poor results. The area under the curve (AUC) of RD and NVC were 0.848 and 0.710, and their combination achieved an AUC of 0.880.

**Conclusion:**

NVC and RD of SpTV are independent risk factors for poor results after MVD surgery, and combining the NVC and RD might achieve relatively high predictive value for poor results.

## Introduction

Trigeminal neuralgia (TN) is the most common functional neurological disorder of the brain, characterized by recurrent episodes of severe pain in the hemifacial trigeminal sensory distribution area, with a prevalence of 22–182 per 100,000, mostly occurring in middle-aged and older adults and seriously affecting physical and mental health [1-5]. The etiology of TN remains unclear, but the prevailing view is that vascular compression and nerve demyelination in the root zone leading to the crosstalk of the nerve conduction signals are the main pathogeneses of TN [2, 5, 6]. Microvascular decompression (MVD) is widely used in clinical practice for treating TN [4, 5]. Compared to other destructive surgical approaches, MVD provides immediate pain relief in about 90% of patients with no neurological impairment and is the surgical treatment of choice for patients without contraindications to anesthesia or surgery [4, 5]. Nevertheless, about 10% of patients show ineffectiveness immediately after MVD surgery, and approximately 20–30% have poor long-term postoperative results [1]. Moreover, MVD has inherent risks and potential complications, including cranial injuries, such as cranial nerve palsy (4%), hearing loss (1.8%), and facial hypoesthesia (3%), and severe complications, such as death (0.3%), stroke (0.6%), and meningitis (0.39%) [3].

Therefore, predicting the results of MVD preoperatively is important to avoid unnecessary cranial injuries. Patients with typical facial pain episodes or trigger points have a better prognosis than those with persistent pain or a combined background of persistent pain [5]. Patients showing efficacy with oral carbamazepine have a better prognosis than those without [5, 7-9]. In addition, older and male patients have a better prognosis than female and younger patients [5, 7, 8]. Nevertheless, these indicators are general and are susceptible to subjective judgment; therefore, objective indicators might be more meaningful in assessing the condition.

Conventional structural magnetic resonance imaging (MRI) sequences suggest a better result of MVD for vascular compression combined with morphological changes such as trigeminal root atrophy [10, 11], but approximately 10% of cases with severe neurovascular contact (NVC) do not experience painful symptoms [12-14]. It has been suggested that vascular compression is not the root cause of TN, as there is no difference in the incidence of vascular compression in the TN and normal populations [12]. Diffusion tensor imaging (DTI) is currently the only MRI technique that can detect microstructural changes in vivo and is particularly suitable for changes such as nerve demyelination. In particular, the DTI sequence has been shown to have high sensitivity in detecting microstructural abnormalities across nerve fibers such as trigeminal nerve [15, 16]. Preoperative changes in the trigeminal nerve DTI and its brainstem segment have predictive value for TN treatment response [17]. However, previous studies have typically used regions of interest (ROIs) sampling, and there were differences in sampling locations, resulting in inconsistent results [15-17].

Nociceptive fibers from the trigeminal nerve enter the brainstem and project mainly through the spinal trigeminal tract (SpTV) to the spinal trigeminal nucleus [18]. The characteristics of SpTV fiber bundles extracted using the DTI fiber tracing technique might be used for predicting the results of MVD treatment. Therefore, this study aimed to explore the predictive value of preoperative MRI structural and diffusion indices on the results of MVD in patients with TN. It was hypothesized that ineffective MVD surgery for TN could be related to the microstructural changes at this location.

## Methods

### Study design and patients

This retrospective study included patients diagnosed with typical TN and treated with trans-trigeminal manifest MVD in the Department of Neurosurgery of the Jining First People’s Hospital between January 2020 and January 2021.

The inclusion criteria were (1) symptoms meeting the diagnostic criteria of primary unilateral TN [2], (2) preoperative imaging data and postoperative follow-up data were complete, and (3) at least one outpatient follow-up pain score was recorded within one year after surgery. The exclusion criteria were (1) a history of preoperative trigeminal nerve surgical treatment (including radiofrequency ablation, balloon compression, or gamma knife) or (2) a history of intracranial tumors, multiple sclerosis, or other conditions that may cause secondary TN.

The study was approved by the Ethics Committee of the Jining First People’s Hospital, Shandong Province (Approval number: 062/2021). The requirement of informed consent was waived due to the retrospective nature of this study.

### Data collection and definition

The demographic (including age and gender) and baseline clinical characteristics (including preoperative pain, pain duration, and pain laterality), preoperative MRI, and postoperative data were collected. Postoperatively, pain relief was assessed at least one year after surgery. Pain scores were measured preoperatively and postoperatively using a visual analog scale (VAS) (0 indicates no pain, and 10 indicates the most severe pain imaginable). According to previous studies, those with postoperative facial pain relief ([preoperative VAS score – postoperative VAS score]/preoperative VAS score) of ≥ 75% were defined as good results and included in the good prognosis group; otherwise, they were defined as poor results and included in the poor prognosis group [19]. There was no statistical difference in the follow-up time between the two groups.

All subjects underwent MRI using the following imaging protocol: DTI: Repetition time (TR)/echo time (TE): 10,000 ms/91 ms; field of view (FOV): 256 mm × 256 mm; voxel dimension 2 mm × 2 mm × 2 mm, *b*-value = 0 and 1000 s/mm^2^, collected from 30 directions and a nominal acquisition time of 5.45 min. Three-dimensional T1-weighted imaging: TR/TE: 1900 ms/2.52 ms; FOV: 256 mm × 256 mm; voxel dimension: 1 mm × 1 mm × 1 mm; slice thickness of 1 mm and no gap. Magnetic resonance angiography (MRA): TR/TE: 20 ms/3.59 ms; FOV: 200 mm × 200 mm; slice thickness of 1 mm, resolution 1 mm × 0.5 mm × 0.5 mm. Three-dimensional fast spin echo sequence (SPACE): TR/TE: 1000 ms/135 ms, FOV: 200 mm × 200 mm; slice thickness of 0.5 mm, slice oversampling 20%, matrix: 384 × 384, FA 120°, resolution 0.5 mm × 0.5 mm × 0.5 mm, SNR: 1. All the scans were obtained using a 3 T MRI scanner (Trio system, Siemens, Germany) [20, 21]. The images were analyzed by a senior neuroimaging physician who was blinded to the patient’s results of MVD.

**Fig. 1:**
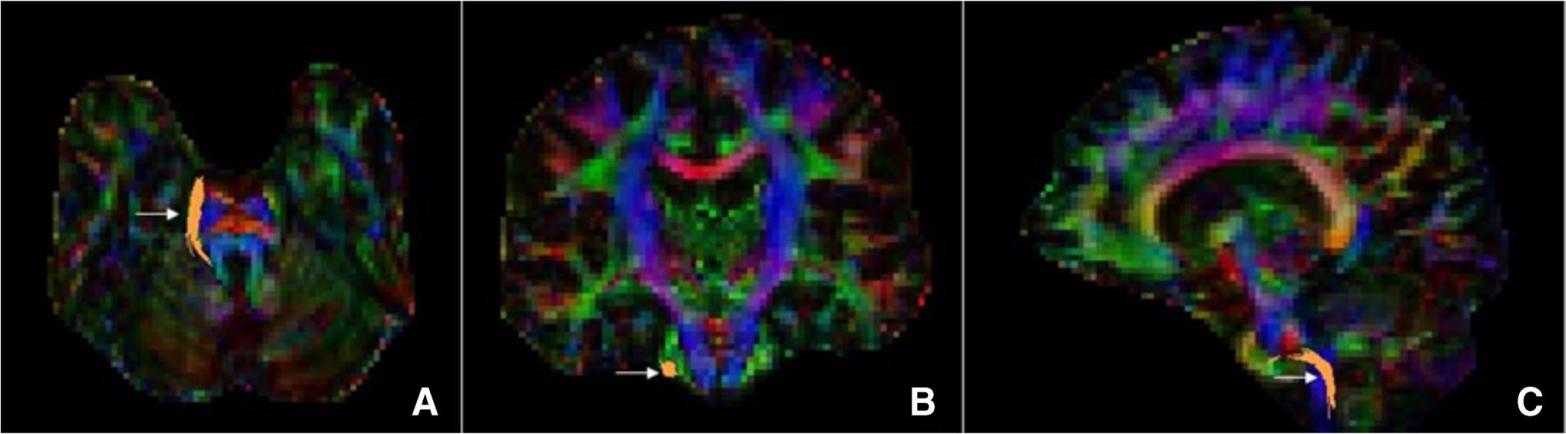
Diffusion tensor imaging tractography of a patient revealed SpTV (white arrows): (**A**) Axial view, (**B**) coronal view, and (**C**) sagittal view

**Fig. 2:**
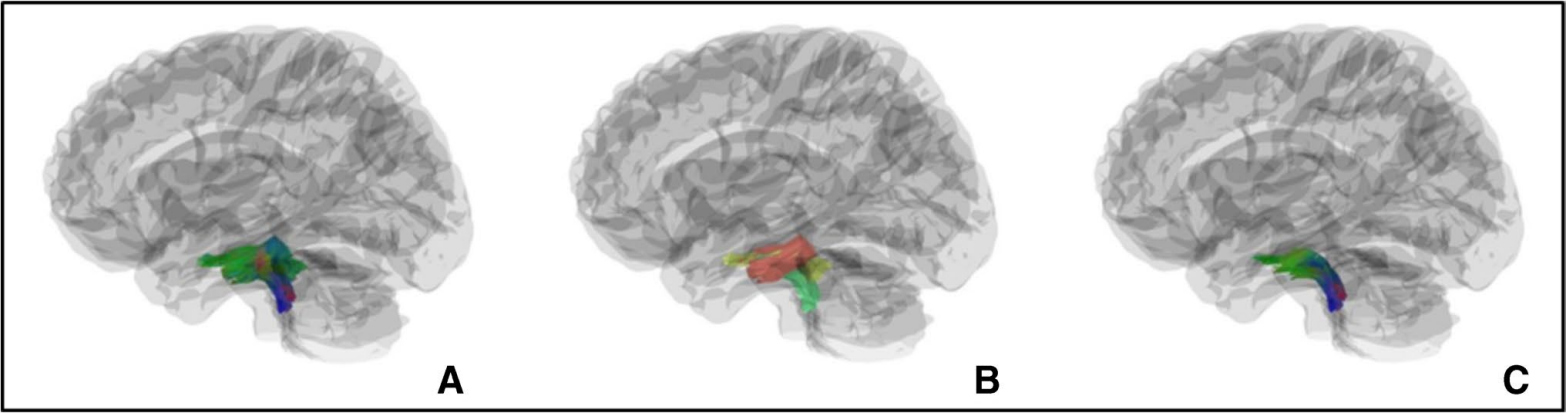
3D reconstruction revealing bundles of SpTV traveling through the brain stem: (**A**) Reconstruction of the whole trigeminal nerve tracts, (**B**) Different colors were used to mark fibers project to different directions, (**C**) The SpTV fibers were identified

The obtained DICOM images were converted to NIfTI format using the dcm2nii program (http://nitrc.org/projects/dcm2nii). Eddy current and motion corrections were performed using FMRIB Software Library (FSL v5.08; http://fsl.fmrib.ox.ac.uk/fsl/) tools. Deterministic tracking of trigeminal fiber bundles was performed using the DSI-Studio software (http://dsi-studio.labsolver.org/) with the same-setting tracking parameters to automate the acquisition of trigeminal fiber bundles. The DTI images were aligned and fused with 3DT1 images of the same patient. SpTV was confirmed by a senior neuroimaging physician under double-blind conditions based on Afshar’s anatomical atlas of the brainstem (Figs. 1 and 2). According to the grading standard proposed by Maarbjerg S, the degree of NVC was observed in three directions (axial, sagittal, and coronal) in the MRA and SPACE sequences, with grade 1 being simple neurovascular contact without compression, grade 2 being contact with nerve distortion, and grade 3 being contact with nerve atrophy [14]. Diffusion indices (including fractional anisotropy [FA] and radial diffusivity [RD]) of the target fiber bundles were automatically acquired by the DSI-Studio software.

### Statistical analysis

SPSS 22.0 (IBM, Armonk, NY, USA) and MedCalc software were used for statistical analysis. Continuous data were presented as means ± standard deviation (SD) and compared by independent samples *t*-test. Categorical data were presented as *n* and compared by the chi-square test. Logistic regression (forward) analysis was performed on variables that were statistically significant in univariate analyses to explore independent risk factors for poor results of MVD. The receiver operating characteristic (ROC) curve was used to assess the diagnostic value of diffusion indexes, and the area under the ROC curve predicted by each indicator was compared using the Delong test. Results were considered statistically significant at two-sided *P* < 0.05.

## Results

### Characteristics of the patients

A total of 97 TN cases were collected, including 43 males and 54 females, 47 on the left side and 50 on the right side. A total of 73 patients were included in the good results group and 24 in the poor results group. There were no statistically significant differences in gender, age, follow-up time, pain score, pain duration, and pain laterality at baseline between the two groups (Table 1).

**Table 1:** Comparison of baseline information between the poor and good prognosis groups

Characteristics	Poor prognosis (*n* = 24)	Good prognosis (*n* = 73)	P
Sex (male/female)	24 (10/14)	73 (33/40)	0.762
Age (mean ± SD), years	53.2 ± 11.4	55.2 ± 9.5	0.390
Pain duration (mean ± SD), years	6.0 ± 2.0	6.6 ± 1.8	0.157
VAS score (mean ± SD)	6.2 ± 2.3	6.0 ± 2.0	0.751
Pain laterality (left/right)	24 (13/11)	73 (34/39)	0.519
Follow-up time (mean ± SD), months	16.08 ± 3.71	16.82 ± 3.69	0.887

*VAS*, visual analog scale

### Risk factors for poor results

FA was significantly lower (*P* < 0.001), while RD of SpTV was significantly higher (*P* < 0.001) in the poor results group compared to the good results group. There was a statistical difference in the distribution of the NVC scores between the two groups, with the poor results group showing a lower proportion of grade 3 NVC (16.7% vs. 39.7%, *P* = 0.001) (Table 2).

**Table 2:** Comparison of imaging indicators between the poor and good prognosis groups

Characteristics	Poor prognosis (*n* = 24)	Good prognosis (*n* = 73)	*P*
FA (mean ± SD)	0.40 ± 0.04	0.44 ± 0.05	< 0.001
RD (mean ± SD) 10^−3^mm^2^/s	1.06 ± 0.14	0.87 ± 0.12	< 0.001
NVC grade			0.001
1	13	12	0.001
2	7	32	
3	4	29	

*FA*, fractional anisotropy; *RD*, radial diffusivity; *NVC*, neovascular contact

The multivariate analysis of the prognostic impact of preoperative imaging indicators showed that the RD of SpTV (OR = 0.000016, 95% CI: 0.000–0.004, *P* < 0.001) and NVC (OR = 8.07, 95% CI: 1.67–38.93, *P* = 0.009) were independently associated with poor results of MVD (Table 3).

**Table 3:** Logistic multivariable analysis of the prognostic impact of preoperative imaging indicators

Characteristics	OR (95%CI)	P
RD (10^−3^mm^2^/s)	0.000016 (0.000–0.004)	< 0.001
NVC grade		
1	1.00	
2	3.503 (0.901–13.263)	0.070
3	8.070 (1.673–38.930)	0.009

*RD*, radial diffusivity; *NVC*, neovascular contact

### ROC analysis

**Fig. 3:**
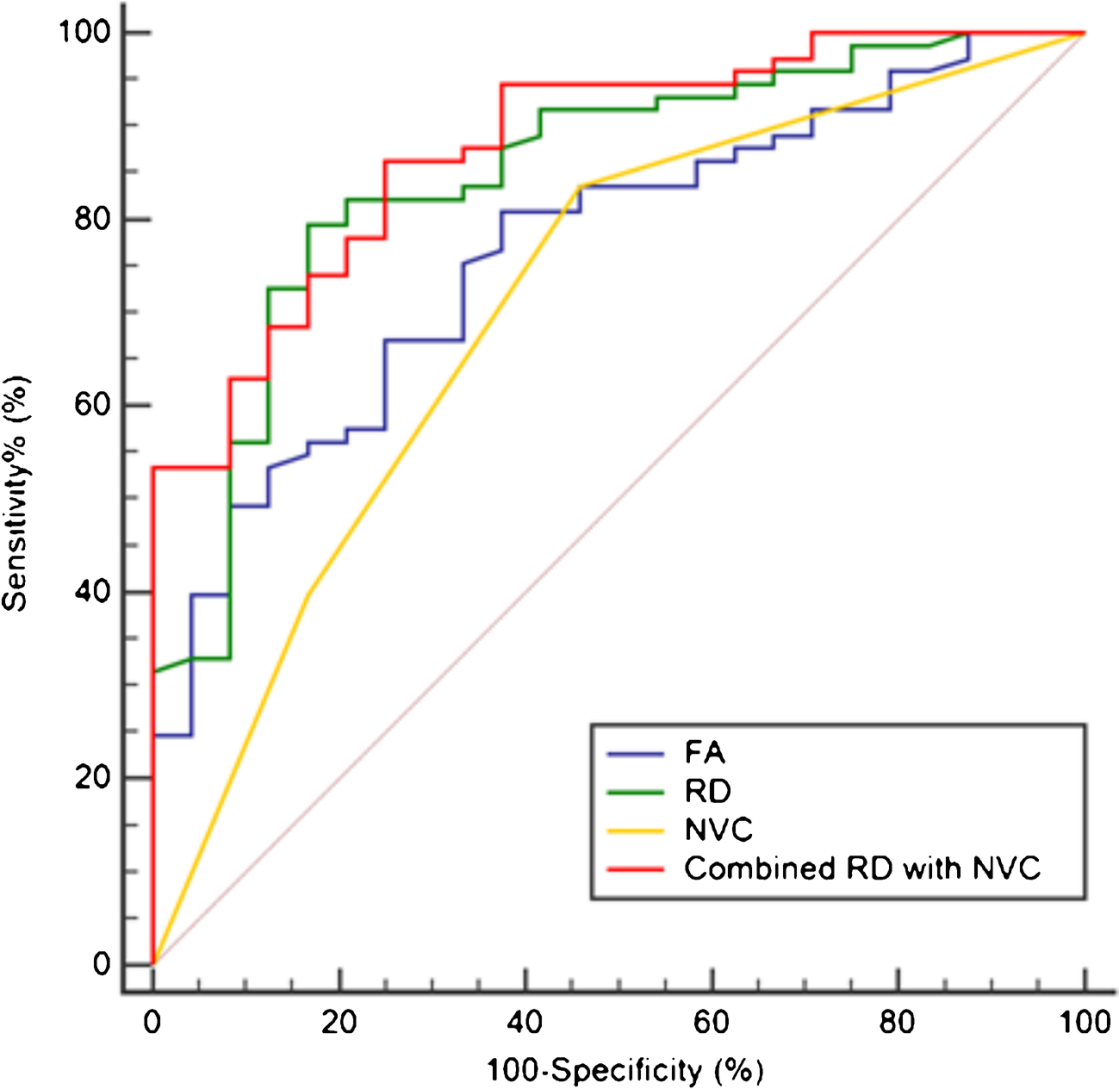
ROC curves of different predictive indicators for MVD results

The AUC of combined diagnosis including RD and NVC was significantly higher compared to that of NVC (0.880 vs. 0.710, DeLong test, *P* < 0.001) (Table 4 and Fig. 3). However, there was no statistical difference between the AUC of combined diagnosis and RD (0.880 vs. 0.848, Delong test, *P* > 0.05). The optimal cut-off value of RD was 0.9782 × 10^−3^mm^2^/s, achieving a sensitivity of 83.3% (95% CI, 62.6–95.3%) and a specificity of 79.4% (95% CI, 68.4–88.0%).

**Table 4:** Predictive value of risk factors for poor prognosis after MVD

Characteristics	AUC	OR (95%CI)	*P*
RD (10^−3^mm^2^/s)	0.848	0.761 to 0.913	0.000
NVC grade	0.710	0.609 to 0.798	0.001
Combined diagnosis	0.880	0.798 to 0.937	< 0.001

*AUC*, area under the curve; *OR*, odds ratio; *CI*, confidence interval; *RD*, radial diffusivity; *NVC*, neovascular contact

## Discussion

This study suggested that NVC and RD of SpTV are independent risk factors for poor results after MVD surgery, and combining NVC and RD might achieve relatively high predictive value. The result could help improve the efficacy of predicting the prognosis of TN surgery.

In the present study, the SpTV was successfully extracted. The trigeminal nerve fibers through the SpTV are mainly composed of nociceptive fibers. The microstructural analysis of the SpTV is necessary to understand the pathogenesis of TN and the causes of ineffective MVD [22].

The analysis of the diffusion indices of the fiber bundles can improve the reliability of the results [23]. The trigeminal nerve fibers are complex in composition, and their sensory roots enter the brainstem and project to the midbrain, the pontine sensory principal nucleus, the medulla oblongata, and the spinal tract nuclei of the superior cervical medulla, respectively [24]. The fiber bundles have long travel distances, making it difficult to overcome manual sampling errors by manually selecting areas of interest for sampling, and the results do not reflect the changes in the fiber bundles as a whole. The trigeminal nociceptive fibers are composed of Aδ and C fibers that enter the brainstem and project downwards in an “elbow” shape to the spinal trigeminal nucleus in the medulla [25]. In this study, the DSI-studio software, which is robust for brain nerve tracing, was used to automate the tracing and extraction of the trigeminal nerve fiber bundles and obtain the SpTV fibers, which were spatially aligned to match the anatomical atlas of the brainstem. Burkett et al. [26] used DTI deterministic tracing to successfully trace these fiber bundles in 20 subjects, and comparison with the anatomical brainstem atlas proved that the alignment was reasonable, but no diffusion index was obtained, and no further diffusion index differences were analyzed. The tracking parameters we used were in general agreement with those set by Burkett et al. [26], and the ability to obtain diffusion values was expected to reduce the bias of manual selection and increase the accuracy and usefulness of the measurements.

Abnormalities in the brainstem segment of the trigeminal nerve are associated with poor prognosis in TN treatment [1], but the results of studies on the correlation between trends in diffusion indexes and the prognosis of TN treatment vary considerably from center to center. Indeed, lower FA and higher RD in the entry zone and brainstem were associated with a poor prognosis in TN [19, 27]. In contrast, Willsey et al. [28, 29] concluded that lower FA and higher RD in the root entry zone and brainstem of the trigeminal nerve fiber bundles were characteristic of the TN1 type patients and predicted a better surgical prognosis. The present study showed a decrease in FA and an increase in RD in SpTV of TN patients who did not respond to MVD treatment, which was in general agreement with Chen et al. [30], although the method used in the present study is probably more reproducible, as the quantitative nerve parameters were extracted from the automated nerve tracing and not from the manual segmentation of the ROIs. FA is the most frequently used index to evaluate nerve fiber integrity and represents the degree of anisotropy. RD indicates the diffusion rate perpendicular to the axonal fibers and reflects the integrity of the myelin sheath, and its elevation may reflect myelin damage [31]. Therefore, impaired nociceptive fibers might be present in this group of patients. It is important to note that SpTV is dominated by unmyelinated C-fibers, and the microstructural changes reflected by the above diffusion index abnormalities and their corresponding pathological changes need to be further investigated.

The NVC score is a common clinical predictor of MVD prognosis and is widely used in clinical practice to guide the development of treatment plans. Its disadvantage is that it only reflects extrinsic nerve compression. Clinically, TN without nerve compression and vice-versa in normal people are both common, and the diagnostic specificity and sensitivity of NVC are limited. Therefore, other indicators need to be combined with NVC in clinical practice. In the present study, the AUC of the ROC curve predicted by NVC alone was 0.710, but when combined with RD of the SpTV, which is the main trigeminal nociceptive fiber, its predictive efficacy was greatly improved, with a combined diagnostic AUC of 0.880, indicating the feasibility of this method. A previous study suggested that combining clinical features such as the type of pain and the effectiveness of oral carbamazepine might further improve predictive efficacy [12]. Since this study was retrospective, it did not allow for an accurate review and statistics of preoperative pain traits. In addition, clinical observation was typically recommended for patients with ineffective oral carbamazepine treatment.

This study had some limitations. First, it was a retrospective study of short duration without a long follow-up, reducing its accuracy. The predictive value of long‐term clinical outcomes could not be assessed due to a lack of follow-up on long-term efficacy. Second, images were analyzed by one investigator, but ideally, neuroradiologists should have analyzed the images, followed by Cohen’s kappa coefficient calculation. Moreover, since the SpTV analyzed in this study were mainly unmyelinated fibers, axial diffusivity (AD) was not included as an indicator of myelin integrity. It is undeniable that AD is very meaningful in the study of changes in the microstructure of nerve fibers. We believe reanalysis of this indicator is warranted as the number of cases accumulates in future studies. And then, the diagnostic efficacy of the DTI index was high, but the RD units were small, and the difference between normal and abnormal values was not significant. There is a risk of error in the lateral comparison of data obtained on different machines or with different scanning parameters. The next step would be setting uniform scanning parameters for this group of patients, which could facilitate the clinical application of the analysis of DTI. Alternatively, the non-symptomatic side could be used as a reference, for example, by taking the difference between the diffusion index on the symptomatic side and the non-symptomatic side. Finally, the OR of RD was very small, and the discrimination in actual comparison is not good. It might be because the RD units were small, and the sample size of patients with poor results was limited in this study, while the ROC of combined diagnosis according to multivariate analysis showed good predictive value.

In summary, NVC and RD of SpTV are independent risk factors for poor results after MVD surgery, and combining the NVC and RD might achieve relatively high predictive value for poor results.

## Data Availability

The datasets used and/or analyzed during the current study are available from the corresponding author on reasonable request.

## References

[1] Cruccu G, Di Stefano G, Truini A (2020). Trigeminal Neuralgia. N Engl J Med.

[2] Headache Classification Committee of the International Headache Society (IHS) (2018) The International Classification of Headache Disorders, 3rd edition. Cephalalgia 38:1-211 10.1177/0333102417738202 29368949

[3] Bendtsen L, Zakrzewska JM, Abbott J (2019). European Academy of Neurology guideline on trigeminal neuralgia. Eur J Neurol.

[4] Zakrzewska JM, Linskey ME (2015). Trigeminal neuralgia. BMJ.

[5] Maarbjerg S, Di Stefano G, Bendtsen L, Cruccu G (2017). Trigeminal neuralgia – diagnosis and treatment. Cephalalgia.

[6] Love S, Coakham HB (2001). Trigeminal neuralgia: pathology and pathogenesis BRAIN.

[7] Bick SK, Huie D, Sneh G, Eskandar EN (2019). Older patients have better pain outcomes following microvascular decompression for trigeminal neuralgia. Neurosurgery.

[8] Ko AL, Lee A, Raslan AM, Ozpinar A, McCartney S, Burchiel KJ (2015). Trigeminal neuralgia without neurovascular compression presents earlier than trigeminal neuralgia with neurovascular compression. J NEUROSURG.

[9] Di Stefano G, La Cesa S, Truini A, Cruccu G (2014). Natural history and outcome of 200 outpatients with classical trigeminal neuralgia treated with carbamazepine or oxcarbazepine in a tertiary centre for neuropathic pain. J HEADACHE PAIN.

[10] Hughes MA, Jani RH, Fakhran S et al (2019) Significance of degree of neurovascular compression in surgery for trigeminal neuralgia. J Neurosurg 1–6 10.3171/2019.3.JNS183174 31200377

[11] Heinskou TB, Rochat P, Maarbjerg S (2019). Prognostic factors for outcome of microvascular decompression in trigeminal neuralgia: a prospective systematic study using independent assessors. Cephalalgia.

[12] Burchiel KJ (2016). Trigeminal neuralgia: new evidence for origins and surgical treatment. Neurosurgery.

[13] Jani RH, Hughes MA, Gold MS, Branstetter BF, Ligus ZE, Sekula RJ (2019). Trigeminal nerve compression without trigeminal neuralgia: intraoperative vs imaging evidence. Neurosurgery.

[14] Maarbjerg S, Wolfram F, Gozalov A, Olesen J, Bendtsen L (2015). Significance of neurovascular contact in classical trigeminal neuralgia. Brain.

[15] Herweh C, Kress B, Rasche D (2007). Loss of anisotropy in trigeminal neuralgia revealed by diffusion tensor imaging. Neurology.

[16] Lutz J, Thon N, Stahl R (2016). Microstructural alterations in trigeminal neuralgia determined by diffusion tensor imaging are independent of symptom duration, severity, and type of neurovascular conflict. J NEUROSURG.

[17] Hung PSP, Chen DQ, Davis KD, Zhong J, Hodaie M (2017). Predicting pain relief: use of pre-surgical trigeminal nerve diffusion metrics in trigeminal neuralgia. NeuroImage Clinical.

[18] Patel NM, Jozsa F, M DJ (2022) Neuroanatomy, Spinal Trigeminal Nucleus 30969551

[19] Tohyama S, Hung PS, Zhong J, Hodaie M (2018). Early postsurgical diffusivity metrics for prognostication of long-term pain relief after Gamma Knife radiosurgery for trigeminal neuralgia. J NEUROSURG.

[20] Li R, Chang N, Liu Y (2021). The integrity of the substructure of the corpus callosum in patients with right classic trigeminal neuralgia. J CRANIOFAC SURG.

[21] Li R, Sun H, Hao H (2021). White matter integrity in patients with classic trigeminal neuralgia: a multi-node automated fiber tract quantification study. J INT MED RES.

[22] Wilcox SL, Gustin SM, Macey PM, Peck CC, Murray GM, Henderson LA (2015). Anatomical changes at the level of the primary synapse in neuropathic pain: evidence from the spinal trigeminal nucleus. J NEUROSCI.

[23] Zhang F, Xie G, Leung L (2020). Creation of a novel trigeminal tractography atlas for automated trigeminal nerve identification. Neuroimage.

[24] Terrier LM, Hadjikhani N, Destrieux C (2022). The trigeminal pathways J Neurol.

[25] DaSilva AF, DosSantos MF (2012). The role of sensory fiber demography in trigeminal and postherpetic neuralgias. J DENT RES.

[26] Burkett DJ, Garst JR, Hill JP, Kam A, Anderson DE (2017). Deterministic tractography of the descending tract of the spinal trigeminal nerve using diffusion tensor imaging. J NEUROIMAGING.

[27] Tohyama S, Walker MR, Zhang JY, Cheng JC, Hodaie M (2021). Brainstem trigeminal fiber microstructural abnormalities are associated with treatment response across subtypes of trigeminal neuralgia. Pain.

[28] Willsey MS, Collins KL, Conrad EC, Chubb HA, Patil PG. (2019) Diffusion tensor imaging reveals microstructural differences between subtypes of trigeminal neuralgia. J Neurosurg 1–7 10.3171/2019.4.JNS19299 31323635

[29] Willsey MS, Mossner JM, Chestek CA, Sagher O, Patil PG (2021). Classifier using pontine radial diffusivity and symptom duration accurately predicts recurrence of trigeminal neuralgia after microvascular decompression: a pilot study and algorithm description. Neurosurgery.

[30] Chen TY, Ko CC, Wu TC (2021). Longitudinal alterations of the cisternal segment of trigeminal nerve and brain pain-matrix regions in patients with trigeminal neuralgia before and after treatment. BMC NEUROSCI.

[31] Chen DQ, DeSouza DD, Hayes DJ, Davis KD, O'Connor P, Hodaie M (2016). Diffusivity signatures characterize trigeminal neuralgia associated with multiple sclerosis. Mult Scler.

